# Successful endovascular thrombectomy of a dislodged hemodialysis catheter fragment and thrombus from the superior vena cava via the basilic vein: A case report

**DOI:** 10.1016/j.radcr.2025.05.075

**Published:** 2025-06-19

**Authors:** Charlotte Wintergerst, Katharina Vogt, Michael Doppler, Eric Peter Prager, Wibke Uller, Elif Can

**Affiliations:** a Department of Diagnostic and Interventional Radiology, Faculty of Medicine, Medical Center University of Freiburg, University of Freiburg, Freiburg, Germany; b Department of Nephrology, Faculty of Medicine, Medical Center University of Freiburg, University of Freiburg, Freiburg, Germany

**Keywords:** ABO-incompatible kidney transplantation, Thrombectomy, Tunneled hemodialysis catheter, Dialysis, Interventional radiology

## Abstract

In multimorbid patients with end-stage kidney disease awaiting renal transplantation, thromboembolic events may significantly complicate and delay the transplantation process. In the period bridging the transplant, hemodialysis is often carried out using a hemodialysis catheter, which is susceptible to complications. We report a case of a 43-year-old woman with Anti-Neutrophil Cytoplasmic Antibodies negative vasculitis, awaiting an ABO-incompatible kidney transplant, and whose hemodialysis catheter required removal due to improper functioning. Computed tomography revealed the presence of a persistent dislodged fragment of the hemodialysis catheter, accompanied by thrombus formation in the superior vena cava and internal jugular vein. We successfully performed endovascular, minimally invasive thrombectomy via right basilic vein access, removing thrombus and dislodged catheter fragments from the superior vena cava. We then reimplanted a new tunneled hemodialysis catheter. This case underscores the feasibility and clinical benefits of targeted interventional techniques in managing complex complications in patients with end-stage kidney disease.

## Background

Patients with end-stage renal disease (ESRD) face a 2.3-fold higher risk of venous thromboembolic events compared to the general population [1]. Although no standardized treatment algorithm exists for central venous thrombosis, management typically involves anticoagulation [2]. Pretransplant care for patients with ESRD presents significant challenges, particularly when balancing the risks and benefits of dual antiplatelet therapy. Renal transplantation is often deferred in these cases if the risks of discontinuing antiplatelet therapy outweigh the potential benefits of proceeding with the transplant [3]. Delaying necessary kidney transplantation for an extended period poses significant risks to the patient's overall health and clinical outcomes [1]. One bridging therapy before transplantation is the implantation of a tunneled hemodialysis catheter; however, complications from these catheters may occur [4].

The development of mechanical thrombectomy devices has introduced a promising approach for treating thrombosis through the direct mechanical removal of thrombotic material. Although primarily utilized for deep vein thrombosis in the iliac veins, mechanical thrombectomy has been reported as a feasible option for treating upper central venous thrombosis and removing foreign material [5,6]. We present a case of a successful mechanical thrombectomy of dislodged parts of a tunneled hemodialysis catheter and thrombotic material from the superior vena cava (SVC) via the basilic vein in a patient with ESRD awaiting kidney transplantation.

## Case presentation

A 43-year-old woman was admitted to a referral center on October 22, 2024, for the preparation of an ABO-incompatible kidney donation by her husband. Her history was notable for ANCA (Antineutrophil Cytoplasmic Antibodies) -negative vasculitis with renal involvement, resulting in dialysis dependency since June 2022. She had been undergoing hemodialysis 4 times weekly using a tunneled hemodialysis catheter. The patient had no clinical signs of systemic infection and reported no respiratory symptoms or abdominal discomfort at admission. Laboratory analysis at admission showed an elevated C-reactive protein of 50 mg/L, which was attributed to soft tissue calcifications in the context of a suspected diagnosis of lipocalcinogranulomatosis.

**Fig. 1:**
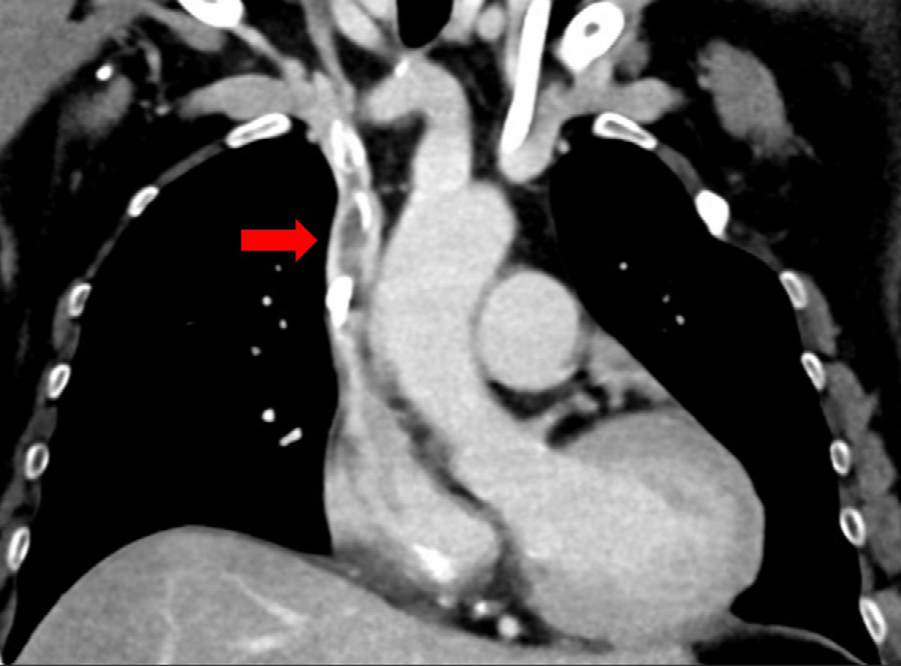
Thrombosis and dislodged catheter parts in the internal jugular vein and superior vena cava. Coronal contrast-enhanced computer tomography reveals a calcified thrombus and dislodged parts of a tunneled hemodialysis catheter in the internal jugular vein (IJV) and superior vena cava (SVC) in a female patient awaiting kidney transplantation due to end-stage renal disease (red arrow).

Relevant comorbidities included hypothyroidism, pulmonary involvement of the ANCA-negative vasculitis with prior hemorrhagic episodes, and a history of arterial hypertension. An earlier placed tunneled hemodialysis catheter via the left internal jugular vein (IJV) for dialysis access was nonfunctional, and there was concern for local infection due to skin erythema at the exit site of the tunneled hemodialysis catheter, necessitating removal and reestablishment of dialysis access. An attempted nontunneled hemodialysis catheter insertion on the right side was unsuccessful. The ultrasound conducted in this context demonstrated thrombosis of the right IJV, which could not be fully traced sonographically in the inferior direction. The subsequent contrast-enhanced computed tomography (CT) revealed the presence of retained catheter remnants in the IJV, extending into the SVC, and a pulmonary embolism involving the right lower lobe pulmonary artery (Fig. 1).

The transplant team decided to postpone the transplantation due to the presence of thrombosis and residual foreign material. In close cooperation between interventional radiology and nephrology, we decided on an interdisciplinary basis that a thrombectomy should be performed, as standard anticoagulation would be insufficient to resolve the issue, particularly due to the presence of retained catheter remnants.

The procedure was scheduled for October 30, 2024, in an angiography suite. The procedure began with an ultrasound of the right upper arm, confirming an adequate vessel diameter. After local anesthesia, we performed ultrasound-guided puncture of the right cephalic vein, with a micro-puncture needle (Micropuncture access set, Cook medical, Bloomington, IN, USA). We advanced a 0.018-inch guidewire through the needle into the SVC, then the needle was exchanged for a micro-puncture sheath. We placed a 0.035″ wire (0.035″ Glidewire, Terumo, Tokyo, Japan) in the right iliac vein. An attempt to exchange the micro-puncture sheath for a 6-French sheath (Prelude Sheath, Merit Medical, South Jordan, UT, USA) resulted in extravasation of contrast material in the upper arm, so we removed all the material and applied focal pressure to the injured cephalic vein. This vessel injury could be conservatively managed without the need for further intervention in the follow-up period. We used the same procedure to perform an ultrasound-guided puncture of the right basilic vein with no complications, and successfully introduced a 6-French sheath (Prelude Sheath, Merit Medical, South Jordan, UT, USA) in the vein.

Using a 0.035″ wire (0.035″ Glidewire, Terumo, Tokyo, Japan), a 5-French Vertebralis catheter (Radifocus™, Terumo, Tokyo, Japan) was advanced to place a long Amplatz wire (Amplatz Super Stiff™, Boston scientific, Marlborough, MA, USA) into the common femoral vein, providing sufficient stability and length for the thrombectomy. The team then introduced a 13-French sheath (ClotTriever Sheath, Inari Medical, Irvine, CA, USA) over the Amplatz wire, allowing for the insertion of the ClotTriever thrombectomy device (Inari Medical, Irvine, CA, USA).

**Fig. 2:**
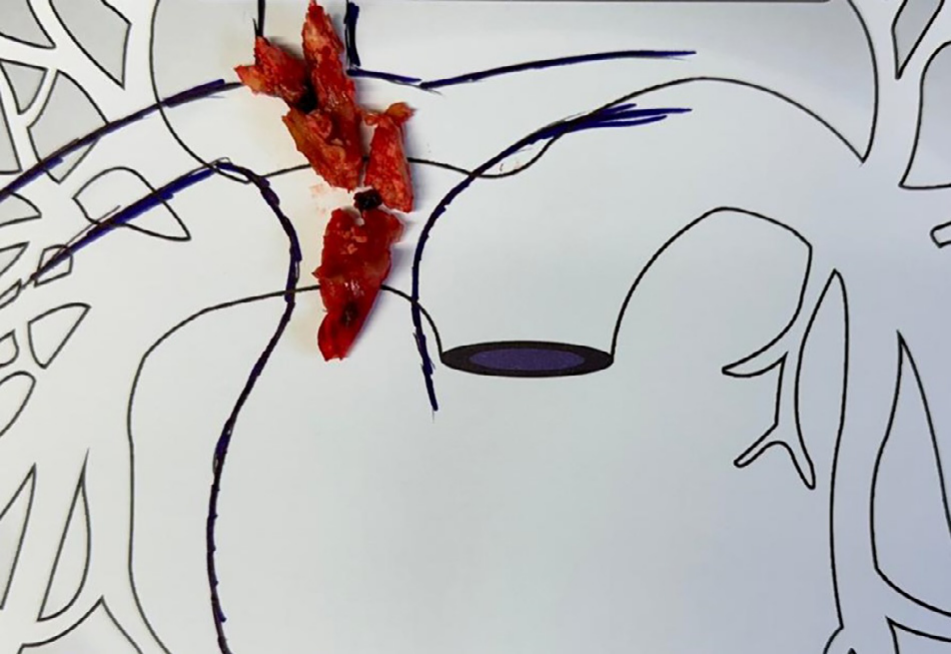
Retrieved thrombus and catheter parts. Retrieved thrombus and dislodged parts of a tunneled hemodialysis catheter following thrombectomy.

Once the ClotTriever catheter was in place, we deployed the expandable nitinol mesh at the site of the thrombus, effectively ensnaring the thrombus material and any associated remnants. The team retraced the device, allowing the efficient removal of the clot material. During the first pass, significant thrombus and remnants of the previous tunneled hemodialysis catheter were extracted (Fig. 2). We followed with a second pass that confirmed no residual thrombus or dislodged catheter fragments remained.

**Fig. 3:**
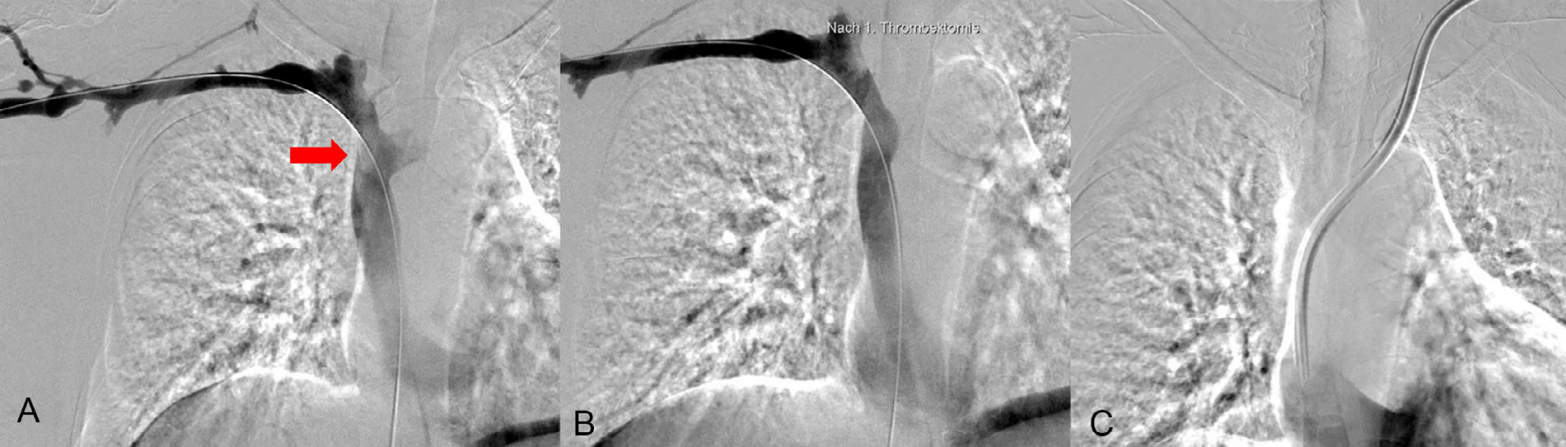
Successful mechanical thrombectomy using a transbasilic access in a 43-year-old female patient awaiting kidney transplantation due to end-stage renal disease. (A) Posteroanterior (PA) digital subtraction angiography (DSA) reveals thrombus and dislodged parts of a tunneled hemodialysis catheter in the superior vena cava (SVC) (red arrow). (B) Following mechanical thrombectomy, both the thrombotic material and any foreign debris are fully removed. (C) We placed a tunneled hemodialysis catheter from the left internal jugular vein (IJV).

After successful thrombectomy using the ClotTriever system, repeat imaging confirmed clearance of thrombus material from the SVC (Fig. 3). The underlying etiology of the thrombus was presumably due to the combination of the patient's prothrombotic risk factors (ANCA-negative vasculitis and ESRD) in conjunction with the presence of a fragmented tunneled hemodialysis catheter. A new tunneled hemodialysis catheter was placed in the left IJV to ensure continued dialysis access before transplantation. The procedure involved ultrasound-guided puncture of the left IJV, placement of a micro-wire, progression to a final catheter with the tip in the right atrium, and subcutaneous tunneling of the distal catheter segment toward the pectoral region.

The ClotTriever system allowed for rapid intervention, with the thrombectomy procedure alone completed within 25 minutes.

**Fig. 4:**
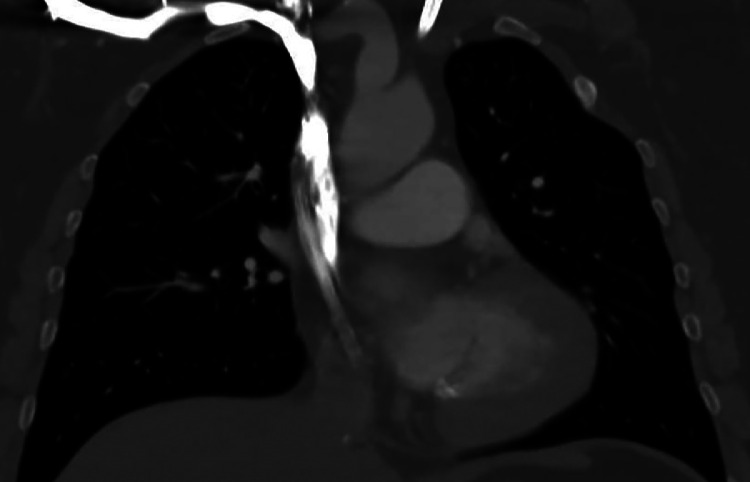
Follow-up 4 months after intervention: no recurrent thrombus in the superior vena cava after mechanical removal of thrombus and dislodged catheter fragments. Coronal contrast-enhanced computed tomography (CT) obtained 4 months after successful mechanical thrombectomy shows restored flow in the superior vena cava (SVC) following removal of thrombus and dislodged catheter fragments. The tunneled hemodialysis catheter via the internal jugular vein (IJV) remains in place.

Postprocedurally, Anticoagulation with heparin was continued, with a transition to enoxaparin at a dose of 4.000 I.U. twice daily for 4 weeks; thereafter, a switch to direct oral anticoagulants was recommended for evaluation. Since postinterventional anticoagulation was to be continued for 6 months, the date of transplantation is set to be 6 months after the thrombectomy. Postoperative follow-up after the intervention confirmed stable hemodialysis catheter function and no evidence of recurrent thrombus formation (Fig. 4).

## Discussion

This case highlights several unique aspects of preoperative management in patients planned for kidney transplantation. Due to the presence of thrombosis, the patient's kidney transplant had to be postponed, which posed a significant risk to her overall health [1]. The decision for minimally invasive thrombectomy was taken in close cooperation between interventional radiology and nephrology, due to the recognition that standard anticoagulation would be insufficient to resolve the issue, particularly due to the presence of retained catheter remnants.

The prompt decision to proceed with thrombectomy, combined with advanced interventional radiology techniques, enabled the rapid and minimally invasive removal of thrombotic material and dislodged components of the tunneled hemodialysis catheter, avoiding the risks associated with general anesthesia. The ClotTriever system enabled fast and effective clot removal, while also addressing the remnants of an old tunneled hemodialysis catheter that would have otherwise been a persistent source of thrombosis and possible infection. With this minimally invasive intervention, we could restore adequate venous access, enabling the implantation of a new tunneled hemodialysis catheter.

A series of case reports have demonstrated that mechanical thrombectomy with the ClotTriever system is a feasible option for treating thrombi in the upper extremity veins and upper central veins [5,7]. Our case demonstrated for the first time that this approach not only allows for the removal of thrombotic material but also enables the retrieval of dislodged catheter parts in the SVC via the basilic vein in a multimorbid patient population.

For the minimally invasive thrombectomy with the ClotTriever device, a large-bore sheath (>13-French) is required. The manufacturer recommends access through veins with a diameter greater than 6 mm. Previous case reports successfully reported the use of the ClotTriever thrombectomy device via upper extremity and central venous access [5,7]. In our patient, after ultrasound-guided puncture and wire advancement, an initial attempt to introduce a 6-French sheath into the cephalic vein (>6 mm in ultrasound) resulted in venous injury and extravasation of contrast material. This complication could be managed conservatively by applying focal pressure. Patients with ESRD have altered vascular properties due to endothelial dysfunction, which is defined as impaired endothelium-dependent vasodilation, increased platelet and leukocyte adhesion, and decreased nitric oxide production, which is often accompanied by a proinflammatory and prothrombotic state [8]. These altered vascular properties can contribute to complications associated with peripheral venous access when large sheaths are used, even in vessels with an adequate diameter as measured by ultrasound. Interventional radiologists should be aware of the potential risks and complications in this specific patient population.

Through the successful venous catheter management combined with the use of anticoagulation, the patient's clinical status was stabilized, which allowed her pretransplantation preparation to continue. The contribution of the interventional radiology department in this case underscores its role in optimizing preoperative conditions in patients with ESRD, particularly in those with complex vascular access issues.

The case further highlights the necessity for individualized management strategies for thrombosis and close interdisciplinary collaboration to prevent risky delays in planned transplantation.

## Conclusion

This case describes, to our knowledge, the first successful endovascular, minimally invasive thrombectomy via right basilic vein access with the Inari ClotTriever system for dislodged catheter fragment and thrombus extraction in a multimorbid patient with ANCA-negative vasculitis and ESRD awaiting kidney transplantation. This case demonstrates the potential of this minimally invasive approach as well as the unique risks and complications of this patient population.

## Ethics approval and consent to participate

Not applicable.

## Availability of data and materials

Not applicable.

## Author contributions

Conceptualization, supervision, manuscript writing, and review: E.C., C.W.; Procedure performance: C.W., E.C., WU; Imaging analysis, patient care, and follow-up: W.U., M.D., K.V., EP. All authors read and approved the final manuscript.

## Patient consent

Written informed consent for publication of this case report was obtained from the patient.
